# Urinary kallikrein 10 predicts the incurability of gastric cancer

**DOI:** 10.18632/oncotarget.16453

**Published:** 2017-03-22

**Authors:** Takaya Shimura, Masahide Ebi, Tomonori Yamada, Tamaki Yamada, Takahito Katano, Yu Nojiri, Hiroyasu Iwasaki, Satoshi Nomura, Noriyuki Hayashi, Yoshinori Mori, Hiromi Kataoka, Marsha A. Moses, Takashi Joh

**Affiliations:** ^1^ Department of Gastroenterology and Metabolism, Nagoya City University Graduate School of Medical Sciences, Nagoya, Japan; ^2^ Department of Gastroenterology, Aichi Medical University, Nagakute, Japan; ^3^ Department of Gastroenterology, Japanese Red Cross Nagoya Daini Hospital, Nagoya, Japan; ^4^ Okazaki Public Health Center, Okazaki, Japan; ^5^ Vascular Biology Program, Boston Children's Hospital, Boston, MA, USA; ^6^ Department of Surgery, Harvard Medical School and Boston Children's Hospital, Boston, MA, USA

**Keywords:** biomarker, gastric cancer, inoperability, kallikrein 10, urine

## Abstract

The current imaging modalities are not sufficient to identify inoperable tumor factors, including distant metastasis and local invasion. Hence, we conducted this study using urine samples to discover non-invasive biomarkers for the incurability of gastric cancer (GC). Urine samples from 111 GC patients were analyzed in this study. The GC cohort was categorized and analyzed according to disease stage and operability. In the discovery phase, protease protein array analysis identified 3 potential candidate proteins that were elevated in the urine of advanced GC patients compared to early GC patients. Among them, urinary kallikrein 10 (KLK10) was positively associated with tumor stage progression. Moreover, the urinary level of KLK10 (uKLK10) was significantly elevated in the urine of patients with inoperable GC compared to operable GC patients (median, 118 vs. 229; *P*=0.014). The combination of uKLK10, tumor location and tumor size distinguished operability of GC with an area under the curve of 0.859, 82.4% sensitivity and 86.2% specificity. Disease-free survival (DFS) was significantly shorter in GC patients with high uKLK10 compared to those with low uKLK10 (hazard ratio: 3.30 [95% confidence interval, 1.58-6.90] *P*<0.001). Immunohistochemical analyses also demonstrated a positive correlation between tumor stage and KLK10 expression in GC tissues (r=0.426, *P*<0.001). In addition, GC patients with high expression of pathological KLK10 (pKLK10) showed a significantly shorter DFS compared to those with low pKLK10 (hazard ratio: 3.79 [95% confidence interval, 1.27-11.24] *P*=0.010). uKLK10 is a promising non-invasive biomarker for the inoperability and incurability of GC.

## INTRODUCTION

Gastric cancer (GC) is the fourth most common malignancy and the second leading cause of cancer deaths in the world [1]. The standard treatment is endoscopic and/or surgical resection for operable stage I-III GC. However, surgical resection is not indicated for unresectable local advanced GC and metastatic GC, and neoadjuvant or palliative chemotherapy is systemically administered for these conditions. Preoperative staging diagnosis is thus very important for choosing an optimal treatment for GC.

Contrast-enhanced computed tomography (CECT) has generally been used as a diagnostic tool for staging GC for operability. CECT can detect metastasis in the lymph nodes and in distant organs such as the liver and lungs, with tumor size and enhanced intratumor intensity. However, it is difficult to diagnose metastasis when the metastatic tumor size is small [2]. It has been reported that CECT sensitivity, specificity, and accuracy for lymph node metastasis (LNM) detection were 25-51%, 79-92%, and 64-72%, respectively [3, 4]. It is sometimes difficult to judge whether a tumor has invaded into an adjacent organ and is unresectable. Moreover, difficulty with the preoperative diagnosis of peritoneal metastasis is a considerable problem for GC treatment. In general, CECT may provide good accuracy for detecting liver metastasis, but does not have satisfactory diagnostic power for detecting pancreatic invasion, LNM, or peritoneal metastasis. CECT could not correctly diagnose GC in 45% of stage IV patients [5].

^18^F-fluoro-2-deoxyglucose positron emission tomography (FDG-PET) has recently been developed to diagnose GC stage, and is usually integrated with computed tomography (FDG-PET/CT) to provide additional diagnostic power for malignancies [6]. However, FDG-PET/CT did not show additional diagnostic power for LNM compared to CECT where sensitivity, specificity, and accuracy of FDG-PET/CT vs. CECT were 34-41% vs. 51-75%, 88-100% vs. 51-92%, and 51-58% vs. 64-72%, respectively [3, 4]. FDG-PET/CT also showed low sensitivity for peritoneal metastasis [7] and a high detectability of liver and lung metastasis [8], as did CECT. In a previous meta-analysis, sensitivity/specificity for the detection of liver and peritoneal metastasis was, respectively, 54%/98% and 9%/99% on ultrasound, 74%/99% and 33%/99% on CT, and 70%/96% and 28%/97% on FDG-PET/CT [7]. Hence, the quality of current imaging modalities is not sufficient owing to the high false-negative rate. This results in patients with incurable or unresectable GC undergoing unnecessary and costly invasive surgery.

To overcome the limitations of the current imaging modalities, staging laparoscopy has been used to decide an optimal treatment for advanced GC. Staging laparoscopy could improve the diagnostic accuracy of metastasis from 37.8% with CT to 73%, according to a previous study [9]. In particular, staging laparoscopy might be useful for the detection of peritoneal metastasis using direct observation, biopsy, and lavage cytology. Sensitivity, specificity, and accuracy of staging laparoscopy for peritoneal metastasis ranged from 73.7-100%, 83-100%, and 93.4-100%, respectively, in a previous systemic review [10]. However, it is occasionally difficult to detect peritoneal micrometastasis even if peritoneal lavage cytology is performed [11]. Moreover, staging laparoscopy is still invasive with consumption of cost and time.

The serum tumor markers CEA and CA19-9 have sometimes been used in clinical practice; however, they are ineffective for judging operability because of their low sensitivity, even for advanced stage GC (20-50%) [12]. In fact, the latest meta-analysis, which analyzed results from over 5000 GC patients in 46 studies, also reported that the sensitivity of CEA and CA19-9 was only 24.0% and 27.0% for all patients and 39.5% and 44.7% for patients with stage IV disease, respectively [13]. Non-invasive biomarkers that can predict the operability and curability of GC are therefore required. We herein demonstrate the usefulness of an urinary biomarker for detecting incurable GC.

## RESULTS

### Patients

The characteristics of the patients in the present study are shown in Table 1. In total, urine samples of 128 patients, 17 healthy controls and 111 with GC, were analyzed in this study. Among the 111 patients with GC, 94 patients had operable GC of stages I-III and 17 patients had inoperable GC of stages III and IV. Of 17 inoperable GC patients, 2 patients were locally advanced GCs and 15 patients had distant metastases which were observed in 7 at liver, 7 at lymph node, 2 at lung, 4 at peritoneum and 1 at bone. Of 94 operable GCs, only 1 did not undergo surgery due to severe co-morbidity, but the GC of the other 93 patients was successfully resected using endoscopy and surgery. No significant differences were noted for age, sex and serum creatinine level among healthy control, operable and inoperable GCs. In the inoperable GC group, the median primary tumor size was larger and the population included more cardia cancers compared to the operable GC group.

**Table 1:** Characteristics of the study groups

		Gastric Cancer		*P*	Healthy control	*P*
		Operable(N=94)	Inoperable(N=17)		(N=17)	
Age (years)	Median (IQR)	67 (62-73)	72 (65-76)	0.340 ^#1^	68 (63-72)	0.626 ^#4^
Sex	Male	74	12	0.529 ^#2^	12	0.631 ^#3^
	Female	20	5		5	
Serum Cr (mg/dl)	Median (IQR)	0.80 (0.70-0.90)	0.73 (0.60-1.10)	0.967 ^#1^	0.88 (0.67-0.94)	0.906 ^#4^
Tumor size (mm)	Median (IQR)	23 (12-44)	54 (50-70)	<0.001 ^#1^		
Histology, N (%)	Intestinal type	70 (74.5)	10 (58.8)	0.240 ^#2^		
	Diffuse type	24 (25.5)	7 (41.2)			
Location, N (%)	Cardia	4 (4.3)	4 (23.5)	0.018 ^#2^		
	Non-cardia	90 (95.7)	13 (76.5)			
Stage, N (%)	I	71 (75.5)	0 (0)	<0.001 ^#3^		
	II	12 (12.8)	0 (0)			
	III	11 (11.7)	2 (11.8)			
	IV	0 (0)	15 (88.2)			
Resection, N (%)	Endoscopy	48 (51.1)	0 (0)	<0.001 ^#3^		
	Gastrectomy	45 (47.9)	1 (5.9)			
	Non-resection	1 (1.1)	16 (94.1)			

N, Number; Cr, Creatinine;

^#1^, the Mann-Whitney U test; ^#2^, Fisher's exact probability test; ^#3^, the χ^2^ test; ^#4^, Kruskal Wallis test.

Clinical stage is according to the seventh edition of UICC-TNM classification.

### Urinary protein array analyses

We performed proteinase protein arrays to identify potential candidate biomarkers involved in GC progression, using 4 urine samples from 2 age- and sex-matched early GC patients and 2 advanced GC patients.

**Figure 1:**
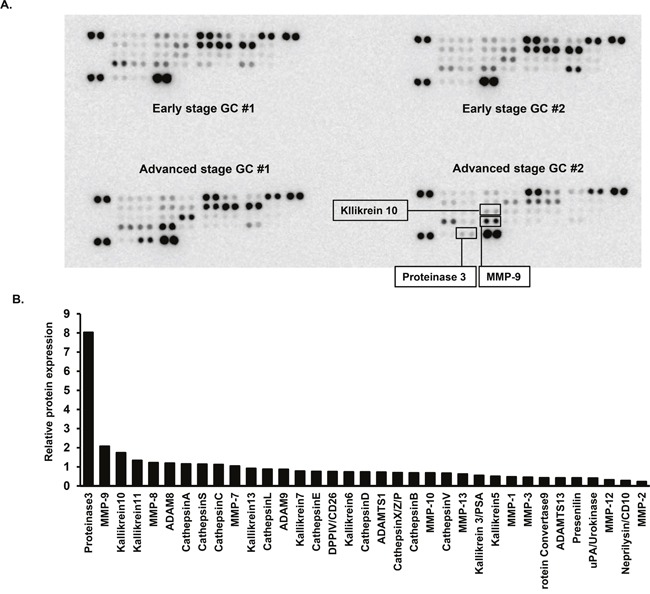
Protease array analyses of urine samples **(A)** Urine samples from 2 patients with early gastric cancer (stage I) and 2 patients with advanced gastric cancer (≥stage III), age- and sex-matched, were used in this analysis. **(B)** Quantification of relative protein expression by densitometry. Bar shows the mean relative protein expression value for 2 advanced gastric cancer patients, compared with 2 early gastric cancer patients.

As shown in Figure 1, the expression levels of proteinase 3 (PRTN3), matrix metalloproteinase 9 (MMP-9), and kallikrein 10 (KLK10) were increased in the urine of advanced GC patients, compared with the urine of early GC patients.

### Enzyme-linked immunosorbent assays

Based on the results from the protease array analyses, we analyzed the urinary protein concentrations of 3 factors, PRTN3, MMP-9, and KLK10, in the whole cohort, using quantitative mono-specific ELISAs. Urinary levels of all proteins were normalized to urinary creatinine (uCr).

**Figure 2:**
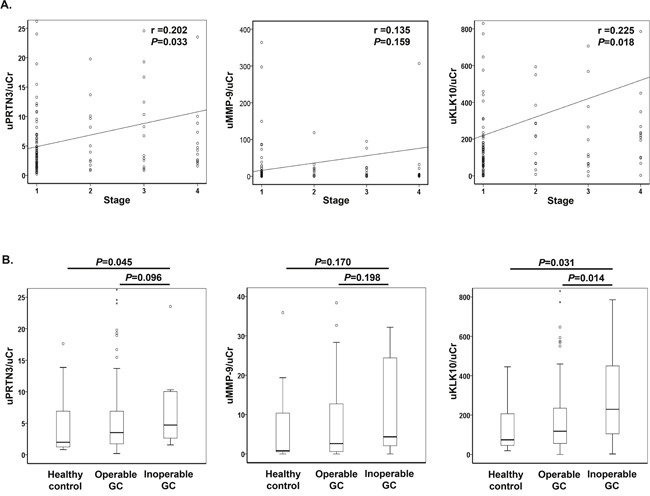
Urinary levels of proteinase 3, matrix metalloproteinase 9 and kallikrein-10 All urinary levels were normalized to urinary creatinine (uKLK10/uCr). **(A)** Correlation between urinary protein level and stage of gastric cancer. Data were analyzed using the Spearman rank correlation. **(B)** Urinary protein levels in healthy control, operable gastric cancer, and inoperable gastric cancer. Data were analyzed using the Mann-Whitney U test. PRTN3, proteinase 3; MMP-9, matrix metalloproteinase 9; KLK10, Kallikrein 10; GC, gastric cancer.

First, we analyzed urinary values of the 3 proteins according to disease stage. Urinary levels of PRTN3 (uPRTN3) and KLK10 (uKLK10) showed a significant positive correlation with disease stage, whereas the urinary level of MMP-9 (uMMP-9) did not (Figure 2A). Next, urinary protein levels were compared between the healthy control, operable GC, and inoperable GC groups. uKLK10 was significantly higher in the inoperable GC group compared to the operable GC group (healthy control vs. operable GC vs. inoperable GC: median uKLK10/uCr, 74 [interquartile range (IQR), 46-207] vs.118 [IQR, 56-235] vs. 229 [IQR, 105-450]), but uPRTN3 was not significant (Figure 2B). Hence, uKLK10 might be considered a biomarker of inoperable GC.

**Figure 3:**
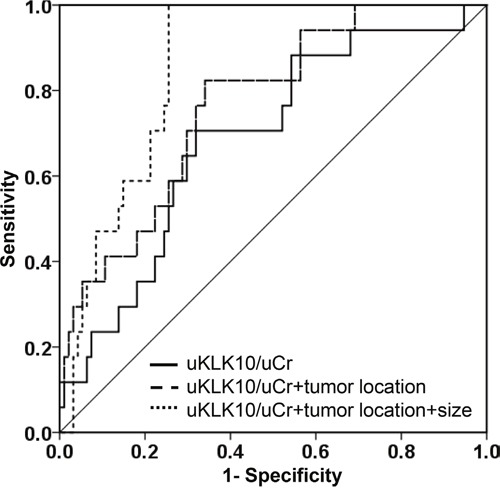
Receiver operating characteristic curves Receiver operating characteristic curves were obtained from normalized urinary kallikrein 10 (uKLK10/uCr), tumor location and tumor size.

Moreover, uKLK10 significantly distinguished between the operable and inoperable GC groups in Receiver operating characteristic (ROC) curve analyses (Figure 3). When tumor location and size, which were significant factors in the inoperable GC groups shown in Table 1, were combined with uKLK10, it demonstrated a great accuracy in distinguishing between operable and inoperable GC (uKLK10: area under the curve (AUC)=0.688 [95% confidence interval (CI), 0.555-0.820], *P*<0.014; uKLK10+tumor location: AUC=0.768 [95% CI, 0.655-0.881], *P*<0.001; uKLK10+tumor location+size: AUC=0.859 [95% CI, 0.789-0.928], *P*<0.001). When the cut-off values were determined as >45 mm tumor size and ≥180 in uKLK10/uCr or cardia GC, inoperable GC could be detected with 82.4% sensitivity and 86.2% specificity.

Of 17 inoperable GC patients of this study, the inoperable tumor factors were found in 5 patients during surgery, which were not detected in the CT scan before surgery (sensitivity: 70.1%). Interestingly, our established combination biomarker could categorize all 5 patients into the inoperable GC group.

### Immunohistochemistry

**Figure 4:**
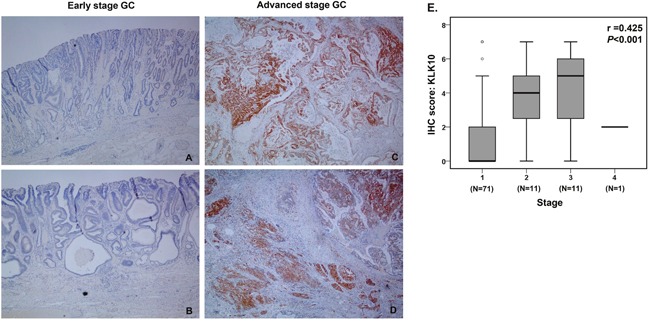
Immunohistochemical analyses of KLK10 **(A-D)** Representative images of KLK10 staining in early and advanced stage gastric cancers (x40). **(A)** No KLK10 expression was detected in early stage gastric cancer tissues (Stage IA, T1bN0M0). **(B)** No KLK10 expression was detected in other early stage gastric cancer tissues (Stage IA, T1aN0M0). **(C)** KLK10 was diffusely and strongly expressed in advanced stage gastric cancer tissues (Stage IIIA: T3N2M0). **(D)** KLK10 diffusely and strongly expressed in advanced stage gastric cancer tissues (Stage IIIC: T4aN3M0). **(E)** Quantitative analysis of immunohistochemistry. Staining scores were calculated in all gastric cancer surgical specimens and data were analyzed using the Spearman rank correlation.

Next, KLK10 expression in GC tissues was analyzed using immunohistochemistry for patients who underwent surgical resection of a primary tumor. Of the 111 GC patients, 94 underwent surgical resection of primary GC. Immunoreactive KLK10 was not detected in most of the early stage GC tissues (Figure 4A, 4B), but it was strongly detected in the advanced GC tissues, and was particularly highly expressed at the invasive front (Figure 4C, 4D). Moreover, immunostaining scores for KLK10 were significantly associated with disease stage (median score, stage I: 0 [IQR, 0-2] vs. stage II: 4 [IQR, 3-5] vs. stage III: 5 [IQR, 3-6] vs. stage IV: 2 [IQR, 2-2]; r=0.426, *P*<0.001) (Figure 4E). Of the patients with stage IV GC, only 1 sample was available for the immunohistochemical study because most of them did not undergo primary resection.

### Disease-free survival

**Figure 5:**
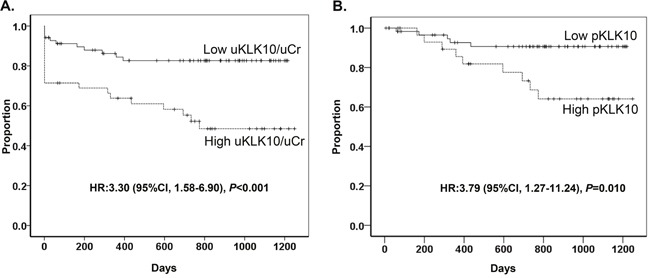
Disease-free survival **(A)** Disease-free survival according to urinal level of KLK10. Urinary level of KLK10 was normalized to urinary creatinine (uKLK10/uCr). **(B)** Disease-free survival according to KLK10 expression in gastric cancer tissue.

According to urinary level and pathological expression of KLK10, disease-free survival (DFS) curves are shown in Figure 5A and 5B. When the GC cohort was categorized into 2 groups according to urinary levels of KLK10, the high uKLK10 group showed a significantly shorter DFS compared to the low uKLK10 group (1y-DFS, 63.8% vs. 84.4%, respectively, *P*<0.001; hazard ratio (HR): 3.30 [95% CI, 1.58-6.90]).

The GC cohort was also divided into 2 groups according to pathological KLK10 expression (pKLK10) in primary gastric tumor tissues: the low pKLK10 group had an immunostaining score of 0-2 while the high pKLK10 group had an immunostaining score of 3-7. The high pKLK10 group had significantly shorter DFS compared to the low pKLK10 group (1y-DFS, 89.3% vs. 92.6%, respectively, *P*=0.010; HR: 3.79 [95% CI, 1.27-11.24]).

## DISCUSSION

In this study, using a variety of biochemical approaches, we have identified uKLK10 as a potential non-invasive biomarker of inoperable and incurable GC. Urine sampling is a straightforward and non-invasive procedure with low risks and costs, making it an attractive option for biomarker detection [14]. Moreover, urinary proteomics approach can reduce the masking effect [15] that obscures detection of low abundant proteins due to high abundant proteins. In fact, we have previously shown the usefulness of urinary biomarkers for the early detection of GC [16], as well as in the diagnosis of other malignancies [17–21]. It has also previously been shown that urinary biomarker protein levels are associated with disease progression in breast cancer [22]. Moreover, urinary biomarkers were useful for predicting the recurrence of some cancers after radiation therapy in another study [23]. Hence, urine is an important and non-invasive sample for the detection of cancer biomarkers.

The human tissue kallikrein and kallikrein-related peptidase (KLK) family has 15 members, making it the largest group of serine proteases [24]. KLKs are secreted proteins and some of those present in body fluids are considered attractive biomarkers for many cancers [24, 25]. The most successful biomarker KLK to date is serum KLK3, which is also called prostate-specific antigen (PSA): serum PSA has been widely used as a diagnostic biomarker for prostate cancer. KLK10, also known as normal epithelial cell-specific 1 (NES1), is a member of the KLK family with a molecular weight of 30 kDa and was originally reported as a tumor suppressor in breast cancer cells [26]. In contrast, KLK10 promoted ovarian cancer cell growth in a recent study [27]. As for human studies, previous studies have demonstrated that mRNA expression of *KLK10* was lower in breast cancer tissues [28], compared to normal tissues, whereas upregulation of KLK10 was associated with a poor prognosis or cancer diagnosis for other cancers including ovarian, pancreatic and colorectal cancers [29–31]. Hence, the true oncological significance and basic mechanism of action of KLK10 remains unclear. In terms of GC, there have been 8 studies that have reported KLK10 as a biomarker [32–39]. Most of these studies showed high expression of KLK10 in cancer lesions, although one study reported downregulation of *KLK10* mRNA expression in GC tissues [37]. All previous reports utilized tissue samples, and analyzed protein and mRNA expression in tumor tissues. However, there have been no reports that detected the presence of circulating KLK10 in the urine and serum of GC patients.

Our study is the first demonstration of the presence of KLK10 in the urine of GC patients. In the present study, KLK10 levels were significantly higher in the urine of inoperable GC patients compared to operable GC patients, and they were positively associated with disease stage, as well as poor DFS. We also analyzed KLK10 protein expression in GC tissues following surgical resection. KLK10 expression in GC tissues was associated with advanced stage and poor DFS. Of the 8 previous studies described earlier in this Discussion, 5 reported that high expression of KLK10 in tumor tissue is associated with advanced stage or poor prognosis [32–35, 38], which supports our findings. In our study, DFS was significantly reduced in GC patients both high level of uKLK10 and pKLK10. This consistency of the result between uKLK10 and pKLK10 may suggest that urinary KLK10 might reflect tumoral expression of KLK10. Since KLK10 is a secreted protein with low molecular weight, urinary measurement might reflect the dynamic KLK10 level in the whole body. Urine is easier and less invasive to collect than tumor tissue. Moreover, and importantly, assessment of pKLK10 would be difficult in biopsy specimens because KLK10 was expressed deep within the invasion margin. A previous study from Grin, et al. also has reported that KLK10 was expressed in the invasive front of GC [32]. Taken together, our use of urinary KLK10 as a biomarker has an advantage over using tumoral KLK10 expression.

There are some reports demonstrating that tumor size is a prognostic factor for GC [40–42], although size was not an independent prognostic factor in other studies [43, 44]. Despite this controversy, tumor size might be a simple and useful parameter that can be easily obtained before treatment. Moreover, cardia GC also seems to be more advances stage and poorer prognosis compared to non-cardia GC [45, 46]. The present study also demonstrated larger tumor size and more frequent cardia cancer in the inoperable GC group than in the operable GC group. The AUC obtained by multiplexing uKLK10, tumor location and size reached to an excellent level of 0.859 for predicting inoperable GC with 82.4% sensitivity and 86.2% specificity. Since our established biomarker correctly categorized 5 inoperable GC patients, who were not detected by CT, into the inoperable group, combination with CT findings may be also good strategy for predicting the inoperability of GC.

The current study has two following limitations. First, healthy controls of the present study were selected from a whole healthy control cohort using age- and sex-matching method. The previous studies have reported that KLK10 is synergistically regulated by steroid hormones such as estrogen, androgen, and progestin [47, 48], and this may influence uKLK10. Case-control method was thus used for the healthy control to compensate the imbalances. Second, the operability was judged by the CT findings in some GCs without surgery, which may arise some biases. However, DFS data of the current study has also demonstrated that uKLK10 contributes to predicting relapse after surgery. Since it is impossible to detect micrometastasis with imaging modality, uKLK10 might be useful to predict the incurability as well as inoperability. In the future, it will be necessary to validate the efficacy of uKLK10 for future clinical applications in a prospective cohort study, as well as to determine oncological mechanisms of KLK10 in GC.

In conclusion, uKLK10 represents a non-invasive biomarker for predicting inoperable and incurable GC, which might help to identify optimal therapeutic and postoperative surveillance strategies.

## MATERIALS AND METHODS

### Patients and study design

All samples were prospectively collected from September 2012 to December 2015 at 3 participating Japanese institutions (Nagoya City University Hospital, Japanese Red Cross Nagoya Daini Hospital, and Okazaki Public Health Center). Patients who met all of the following inclusion criteria were enrolled in this study: aged between 20 and 90 years; histologically confirmed adenocarcinoma using biopsy for the GC group; no treatment before study enrollment for the GC group; and no neoplasms of any type for the healthy control group. Patients with a history of neoplasms of any type and/or with multiple neoplasms were excluded from enrollment in this study. Recurrent GC patients were excluded from this study. The present study included the healthy control cohort for whom age and sex were matched with inoperable GC patients one by one. Age difference within ±5 years was permitted when the sex was the same.

To assure the accuracy and comprehensiveness of reporting this case-control biomarker study, the present study complied with the REMARK guidelines [49] and the STROBE statement [50]. The study protocol was approved by the ethics committee at each institution and was conducted according to the ethical guidelines of the 1975 Declaration of Helsinki (6th revision, 2008). All patients provided written, informed consent before study entry. This study was registered with the University Hospital Medical Information Network Clinical Trials Registry (UMIN000021350).

### Samples and definition

All urine samples were collected before any treatment for GC, immediately frozen and stored at −80°C until assayed, as previously reported [21]. Tissue samples of GC were obtained from primary tumors at the time of the initial surgical and endoscopic resection, fixed in formalin, and embedded in paraffin [51]. Clinical stage was determined by the final pathological diagnosis after resection, according to the 7th edition of the Union for International Cancer Control (UICC) Tumor-Node-Metastasis (TNM) classification [52]. GC with stage IV or unresectable local invasion in the final diagnosis after surgery was defined as the inoperable GC. As for patients without surgery, when distant metastases and severe local invasion were clearly observed by CT scan, it was defined as the inoperable GC.

DFS was defined as the time from the date of cancer diagnosis until the first tumor appearance or death from any cause. In all patients, CT evaluations were performed every 3 to 6 months. The tumor size of GC was defined as the maximum size of the primary tumor on a CT scan at the time of diagnosis.

### Protein array analysis

Since tissue degradation and adhesion by proteases are involved in cancer invasion and metastasis through the cleavage of relevant proteins, we hypothesized that proteases would be potentially useful biomarkers for advanced GC. To screen for urinary proteins in the urine of advanced GC patients, human protease arrays which contain 34 targets were utilized (R&D Systems, Inc., Minneapolis, MN, USA), according to the manufacturer's instructions. We randomly selected 2 early GC patients (stage I) and 2 advanced GC patients (≥stage III) who were completely matched by age and sex, and these 4 urine samples were incubated with antibody-coated array membranes at 4°C overnight. To determine the relative expression level of proteins in advanced GC samples compared to early GC samples, the signal intensities of each protein were quantified by densitometry software, Image Quant TL (GE Healthcare Japan, Tokyo, Japan).

### Enzyme-linked immunosorbent assays

We measured the urinary protein concentration of each of the proteins of interest using mono-specific enzyme-linked immunosorbent assays (ELISAs), according to the manufacturer's instructions. To measure each protein concentration, we used a Quantikine ELISA kit (R&D Systems, Inc.) for MMP-9, a DuoSet ELISA with the DuoSet Ancillary Reagent Kit 2 (R&D Systems, Inc.) for PRTN3, a human kallikrein 10 ELISA kit (RayBiotech, Inc., Norcross, GA, USA) for KLK10, and a Parameter Creatinine Assay (R&D Systems, Inc.) for creatinine. All urine samples were measured in duplicate, and the mean was obtained.

### Immunohistochemistry

Immunohistochemical staining was performed using serial sections of each sample as follows. Consecutive sections (5 μm thick) were deparaffinized and dehydrated through a graded series of xylene and ethanol. After inhibiting endogenous peroxidase activity with 3% hydrogen peroxide (DAKO, Carpinteria, CA, USA) for 5 minutes, blocking of nonspecific binding sites was performed using Protein Block Serum-Free (DAKO) for 20 minutes, according to the manufacturer's instruction. Each section was incubated with a rabbit anti-human KLK10 primary antibody (#bs-2531R: 5 μg/ml, Bioss Inc., Woburn, MA, USA) at 4°C overnight. Samples were then incubated with an anti-rabbit-horseradish peroxidase secondary antibody (DAKO), and immune complexes were visualized by incubation in 3,3′-diaminobenzidine (DAKO). Mayer's hematoxylin was used for nuclear counterstaining.

All immunostained specimens were assessed by an observer blinded to all clinical information. The staining intensity was classified as negative (0), weak (1+), moderate (2+), or strong (3+), and the extent of the staining was defined as the percentage of positive staining areas scored on a scale of 0–4 as follows: 0, 0%; 1, 1-25%; 2, 26-50%; 3, 51-75%; 4, 76-100%, as described in a previous study [53]. The sum of the staining intensity and staining extent scores was used as the final staining score.

### Statistical analyses

The aim of this study was to identify urinary proteins that can diagnose the operability of GC. Quantitative variables were described with the median and IQR and analyzed using the Mann-Whitney U test. Other data were analyzed using the χ2 test or Fisher's exact probability test, as appropriate. The nonparametric Spearman's rank correlation coefficient (r) was used as a measure of correlation. Kaplan-Meier curves were constructed in order to analyze DFS, and differences between the two groups were compared with a log-rank test. Cox proportional hazards model was used to calculate each HR.

The ROC analysis was used to calculate the AUC for each biomarker, and the representative value was shown as the AUC value with 95% CI. The true-positive fractions and the false-positive fractions for diagnosis of GC were calculated for every cut-off level, and the cut-off level was determined by selecting the farthest point from the baseline using ROC analysis. For combinations of biomarkers, the estimated coefficients of a logistic regression model were used to construct a composite score, which was then used to calculate the AUC for the combination of biomarkers. A two-tailed *P* value of less than 0.05 was considered statistically significant.
